# The multispecific thyroid hormone transporter OATP1C1 mediates cell-specific sulforhodamine 101-labeling of hippocampal astrocytes

**DOI:** 10.1007/s00429-013-0645-0

**Published:** 2013-10-16

**Authors:** Christian Schnell, Ali Shahmoradi, Sven P. Wichert, Steffen Mayerl, Yohannes Hagos, Heike Heuer, Moritz J. Rossner, Swen Hülsmann

**Affiliations:** 1 Center for Nanoscale Microscopy and Molecular Physiology of the Brain (CNMPB), Göttingen, Germany; 2 Department of Neurophysiology and Cellular Biophysics, University Medical Center, Göttingen, Germany; 3 Max-Planck-Institute of Experimental Medicine, Research Group “Gene Expression and Signaling”, Göttingen, Germany; 4 Leibniz Institute for Age Research, Junior Research Group “Neuroendocrinology”, Jena, Germany; 5 Department of Systemic Physiology and Pathophysiology, University Medical Center, Göttingen, Germany; 6 Laboratory of Molecular and Behavioral Neurobiology, Department of Psychiatry, Ludwig-Maximilians-University München, Munich, Germany

**Keywords:** Astrocyte, Cell-specific markers, Transcriptome analysis

## Abstract

Sulforhodamine 101 (SR101) is widely used for astrocyte identification, though the labeling mechanism remains unknown and the efficacy of labeling in different brain regions is heterogeneous. By combining region-specific isolation of astrocytes followed by transcriptome analysis, two-photon excitation microscopy, and mouse genetics, we identified the thyroid hormone transporter OATP1C1 as the SR101-uptake transporter in hippocampus and cortex.

**Electronic supplementary material:**

The online version of this article (doi:10.1007/s00429-013-0645-0) contains supplementary material, which is available to authorized users.

## Introduction

The red fluorescent xanthene derivative sulforhodamine 101 (SR101) has been introduced to neuroscience in 1985 as a dye for activity-dependent labeling of synaptic terminals (Lichtman et al. 1985). Since then, different labeling protocols have been used and thus SR101 was shown to stain different cell types in various regions of the brain with considerable species difference. Reports of labeled cell population range from rabbit oligodendrocytes in the retina (Ehinger et al. 1994) to turtle spinal cord neurons (Mui et al. 2012). In 2004, Nimmerjahn and colleagues identified SR101 as a selective marker for cortical astrocytes in vivo after bolus injection or topical application to the cortical surface (Nimmerjahn et al. 2004). Later, SR101 was also confirmed as a valid method in acutely isolated hippocampal and cortex slices (Ikegaya et al. 2005; Kafitz et al. 2008). However, the molecular mechanism of the cell-specific SR101-labeling remained unknown. Initially, it was hypothesized that gap-junction hemichannels were involved in the labeling process (Nimmerjahn et al. 2004); however, astrocytes from Cx30−/−Cx43fl/fl:hGFAP-Cre mice (Cx30−/−Cx43−/−, double knockout) that lack most of the gap-junction protein were effectively labeled with SR101 (Pannasch et al. 2011). Recently, pharmacological profiling of SR101 uptake with inhibitors of organic anion transport led to the conclusion that SR101 was likely to be actively transported into astrocytes via an organic anion-transporting polypeptide (OATP), although the identity of the responsible transporter remained unknown (Schnell et al. 2012).

Furthermore, the use of SR101 has been compromised by recent reports of neuronal SR101-labeling that occurs under pathophysiological conditions such as NMDA receptor activation or ischemia (Thompson et al. 2006; Thompson et al. 2008). Moreover, the side effects of SR101, including chemical LTP, have been reported (Kang et al. 2010; Garaschuk 2013). Therefore, an understanding of the molecular mechanism of SR101 uptake is crucial for its future application as a marker of astrocyte subtypes.

In contrast to the hippocampus, unequivocal identification of astrocytes by SR101 is not possible in the ventral lateral medulla (VLM) of the brainstem, which contains important neurons of the respiratory network. In this region as well as in the rat spinal cord, SR101 has been found to label neurons and astrocytes (Cina and Hochman 2000; Schnell et al. 2012) indicating that a region-specific heterogeneity of astrocytes underlies the lower SR101-uptake efficiencies in the more caudal brain regions.

Based on the pharmacological profile of the SR101-labeling (Schnell et al. 2012) we hypothesize that candidate genes from SLC(O) solute carrier families display elevated mRNA expression levels in forebrain versus brainstem astrocytes. To test for this, we captured EGFP-expressing astrocytes from brainstem (poor SR101 uptake) and hippocampus as well as cortex (both efficient SR101 uptake) from hGFAP-EGFP transgenic mice (Nolte et al. 2001) by fluorescence-activated cell sorting (FACS) and performed digital gene expression profiling using next generation sequencing.

## Materials and methods

### Breeding of mice

Experiments were performed on acute brain slice preparations of transgenic mice [postnatal day 8–43 (P8–P43), male and female] expressing enhanced green fluorescent protein in astrocytes [TgN (hGFAP-EGFP) GFEC-Fki; (Nolte et al. 2001)] or on *Slco1c1* knockout mice (Mayerl et al. 2012). Animals were held and bred in the animal facilities of the University Hospital Göttingen and the Leibniz Institute for Age Research in accordance with guidelines of the German Physiological Society as well as the regulations of the State of Lower Saxony and the Federal Republic of Germany.

### Slice preparations

Acute transversal slices from brainstem and hippocampus were prepared as described previously (Härtel et al. 2007; Schnell et al. 2012). In some experiments, parasagittal slices were cut to compare the SR101 staining along the rostral–caudal extension of the cortex. To obtain acute slices, animals were killed by decapitation under deep diethyl-ether anesthesia. The brains were removed from the skull and the isolated hippocampi and brainstem were placed in ice-cooled, carbogen-saturated (95 % O_2_, 5 % CO_2_) artificial cerebrospinal fluid (aCSF) containing 118 mM NaCl, 3 mM KCl, 1.5 mM CaCl_2_, 1 mM MgCl_2_, 1 mM NaH_2_PO_4_, 25 mM NaHCO_3_, and 30 mM d-glucose. The osmolarity was 325–335 mosm/l and the pH adjusted to 7.4. The isolated brain part was glued with cyanoacryl glue (Loctite Deutschland GmbH) to an agar block and mounted in a vibroslicer (VT 1200S, Leica). Slices of 250–400 μm were cut and stored in oxygenated aCSF at room temperature for at least 30 min before staining. For imaging experiments, slices were transferred to the recording chamber after the staining procedure (see below). Slices were kept submerged by a nylon fiber grid and continuously perfused with aCSF at a flow rate of 5–10 ml/min.

### Sulforhodamine 101 staining protocol

Sulforhodamine 101 (SR101) labeling was performed using the standard protocol as described earlier (Kafitz et al. 2008; Meier et al. 2008; Schnell et al. 2012). Slices were incubated for 20 min at 34 °C in carbogenated aCSF containing 1 μM SR101 followed by 10 min in carbogenated aCSF at 34 °C without SR101 for removal of excess dye from the extracellular space. For the T4/SR101 competition studies, l-Thyroxine (T4; 1–10 μM) was co-applied with SR101 only during the 20 min incubation period.

### Drugs

Electrolytes for aCSF (see above) were purchased from Sigma-Aldrich and Merck chemicals. Drugs were stored in concentrated stock solution at −20 °C. The 1 mM l-thyroxine sodium salt pentahydrate (Sigma-Aldrich; T2501) stock solution was prepared with 0.1 N NaOH and the 0.5 mM SR101 (Sigma-Aldrich, S7635) stock solution was made with distilled water.

### Fluorescence imaging using multifocal two-photon excitation microscopy

For detection of EGFP- and SR101 fluorescence, we used a two-photon microscope (TriMScope, LaVision BioTec) with non-descanned detection by GaAsP photomultipliers (Hamamatsu). Two-photon excitation was achieved with a Ti:Sapphire Laser (SpectraPhysics MaiTai BB) at 800 nm. Fluorescence signals of hGFAP-EGFP expressing astrocytes were detected through a 531/40 nm band pass emission filter, whereas SR101 fluorescence was detected through a 641/75 nm band pass emission filter (AHF Analysentechnik AG). To allow quantitative comparison of the SR101 intensity between controls and drug treatments, all image parameters, pixel dwell time and number, detector gain as well as laser power were identical for a particular set of experiments. Cell counting was performed in a defined volume that was scanned with 2 μm step z-stacks (100 μm in total) using a piezo-focus (Physik Instrumente). All settings were controlled by “Imspector” software (LaVision BioTec).

For quantification of SR101 fluoresence, “Imspector” images were exported to TIFF format. After deconvolution with Autoquant software (MediaCybernetics) using the theoretical point-spread-function (adaptive PSF, ten iterations), further analysis was performed with the Imaris software package (Bitplane) using the “spots” feature of the “surpass” view mode. In this mode, astrocytes were identified by their EGFP fluorescence in the green channel and a spherical 3D volume (spot) of 6 mm diameter was assigned to the soma of each EGFP-positive cell astrocytes.

To quantify the SR101-labeling, the green channel was turned off. The “recentering” function was used to correct the position of the spot in the red SR101 channel. If this was impossible or if the SR101 intensity was not differing from the surrounding background signal, the particular cell was counted as an SR101-negative cell. The parameter “SR101-positive astrocytes (%)” was calculated by dividing the number of both SR101-positive and EGFP-positive cells by the total number of EGFP-positive cells multiplied by 100. SR101- and EGFP-fluorescence intensities of individual cells were derived from the median intensity of the assigned spot and averaged for each slice individually. The parameter “SR101 intensity (% of EGFP fluorescence)” was calculated by dividing the SR101 intensity by the EGFP-fluorescence intensity multiplied by 100.

### Analysis of imaging data

Fluorescence intensity and cell number are presented as means and standard error (SEM). One-way ANOVA tests with multiple comparisons versus control group (Bonferroni *t* test) were used to determine statistical significance, considering *P* ≤ 0.05 as significant. Statistical calculations were performed in SigmaPlot (Systat Software GmbH).

### Fluorescent activated cell sorting (FACS) of EGFP-positive astrocytes

Brains were removed from P10 old hGFAP-EGFP mice (Nolte et al. 2001) and sectioned in oxygenated aCSF at 4 °C with a vibratome to obtain 150 μm thick slices. Cortex, hippocampus and brainstem were subsequently dissected and processed by incubation with freshly prepared Papain solution for 20 min at 37 °C as suggested by the manufacturer (Worthington). Samples were homogenized by gentle trituration with a 1 ml glass pipette, followed by staining of nuclei with cell-permeable nuclear Hoechst 33,342 dye (Sigma) at 37 °C for 10 min. Cells were filtered through 40 μm nylon cell strainer before sorting. FACS isolation of EGFP-positive astrocytes was done using a FACS Aria II (Becton–Dickinson) and gates were set according to control samples for each brain region from wild-type mice. First, forward and side scatter was used to deplete for small sized cellular debris, followed by selection of Hoechst 33,342 and EGFP double-positive cells which were directly sorted into RLT lysis buffer (Qiagen) and frozen at −20 °C until use.

### Amplification of RNA and generation of libraries and Illumina sequencing

For generation of libraries for Illumina sequencing from pools of sorted EGFP-positive astrocytes, we modified an established linear amplification protocol using the DNA-dependent RNA polymerase from bacteriophage T7 (Rossner et al. 2006). Therefore, we first isolated RNA using RNA easy microcolumns (Qiagen) according to manufacturer protocols except of RNA elution, which was done by adding 100 μl H_2_O followed by ethanol precipitation with NH_4_Ac (f.c. 2.5 M) and pellet paint (Invivogen) as carrier. The precipitate was air-dried and resolved in 2 μl of freshly diluted oligo-dT T7-B primer (2 μM). cDNA was synthesized in a total volume of 4 μl using a 2 × pre-mix of Superscript III polymerase (1 h 50 °C) according to manufacturer’s recommendations (Invitrogen). Double-stranded (ds) cDNA was initiated by adding RNAseH and DNA PolI in a volume of 26 μl as described (Rossner et al. 2006). Purification of ds cDNA with GFX columns (GE Healthcare) and T7-mediated antisense RNA amplification (T7 Megascript Kit, Ambion) and aRNA purification were essentially performed as described (Rossner et al. 2006). Second-round cDNA synthesis was initiated with 2 μl of a random hexamer primer (100 μM) with hairpins flanking a sequence stretch for PCR amplification (Dec1-Hairpin-N9), which was shown to suppress false-positive PCR products (Adli et al. 2010) in a volume of 10 μl with Superscript III for 10 min at 25 °C followed by 50 min at 50 °C. RNA was subsequently degraded by adding NaOH (25 min at 68 °C) and neutralized by HCl (1 μl of a 25 mM solution each). Advantage Taq polymerase (Clontech) and primer B-short were used to generate ds cDNA in a volume of 100 μl (according to the manufacturer’s recommendations) and purified using Nucleo-Spin columns (Macherey & Nagel). Pool-identifying barcodes were introduced by PCR using PWO polymerase (Roche) and Code_Dec1 c40 and B-short as primers for ten cycles in a volume of 20 μl and an annealing temperature of 58 °C. PCR products were analyzed by agarose gel electrophoresis and combined to a final sample for the attachment of Illumina sequencing adaptors by five cycles of PCR with PWO (Roche) and 1 μl of each primer (Illumina-for and Illumina-B-rev at 10 μM) in a volume of 100 μl. Size selection (200–400 bp) of the final PCR product was done by agarose gel electrophoresis and gel extraction using the Nucleospin extraction kit (Macherey & Nagel). Sequencing of libraries was performed at the Genome Center Cologne of the Max-Planck-Society according to established protocols on an HiSEQ2000 machine (Illumina) at a depth of 150 Mio reads in total. For primer sequences and graphical representation of the protocol, see supplementary table.

### Processing of reads and bioinformatic analysis

FastQ files were converted into fastA files with the FASTQ-to-FASTA converter and split into pools according to barcodes using the FASTQ/A Barcode splitter (both tools were from http://hannonlab.cshl.edu/fastx_toolkit/), trimmed to 35 bases and mapped to the mouse using the Mus musculus NCBI Built 37.1. Differential expression analysis was performed using the Bioconductor package DESeq (Version 1.4.1) (Anders and Huber 2010).

## Results

### Weak astroglial SR101-labeling in the brainstem

**Fig. 1:**
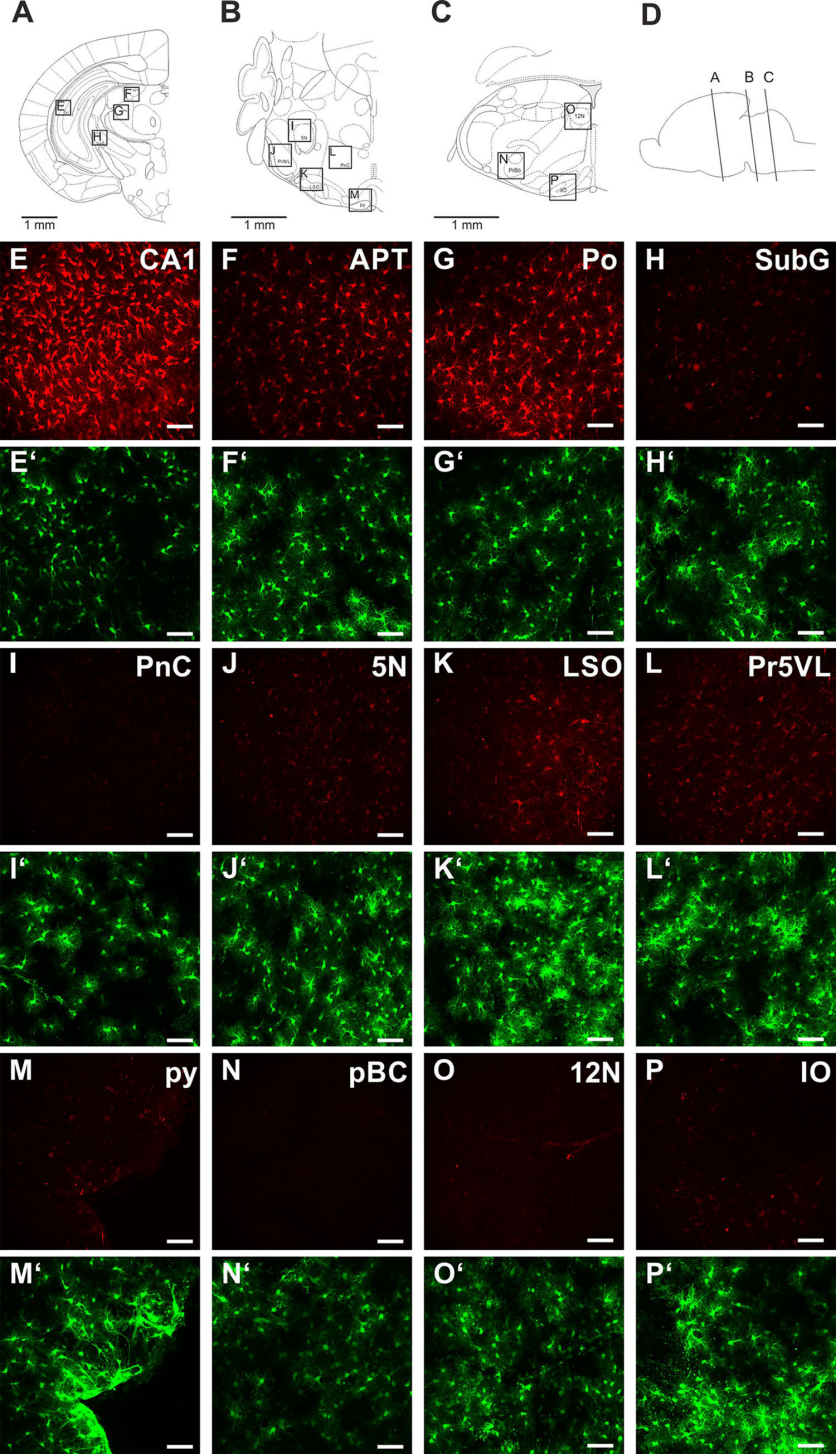
SR101 uptake in various regions of the forebrain and hindbrain. **a**–**c** Coronal diagrams of the brain section analyzed; **d** schematic drawing showing rostro-caudal position of diagrams **a**–**c** (panels **a**–**d** were adapted and modified from the Paxinos Atlas of the developing mouse brain (Paxinos 2007). **e**–**p** Figures are maximal intensity projections of 3D 2-photon scans (100 μm *z*-stack, 51 sections) taken from mice at P8. **e**′–**p**′ The corresponding EGFP expression is shown below the SR101 image. *CA1* corpus ammonis of hippocampus, *APT* anterior pretectal nucleus, *Po* posterior thalamic nuclear group, *PnC* pontine reticular nucleus, caudal part, *5N* motor trigeminal nucleus, *LSO* lateral superior olive, *Pr5VL* principal sensory trigeminal nucleus, ventrolateral part, *py* pyramidal tract, *pBC* pre-Bötzinger complex, *12N* hypoglossal nucleus, *IO* inferior olivary nucleus. *Scale bars* 40 μm

**Fig. 2:**
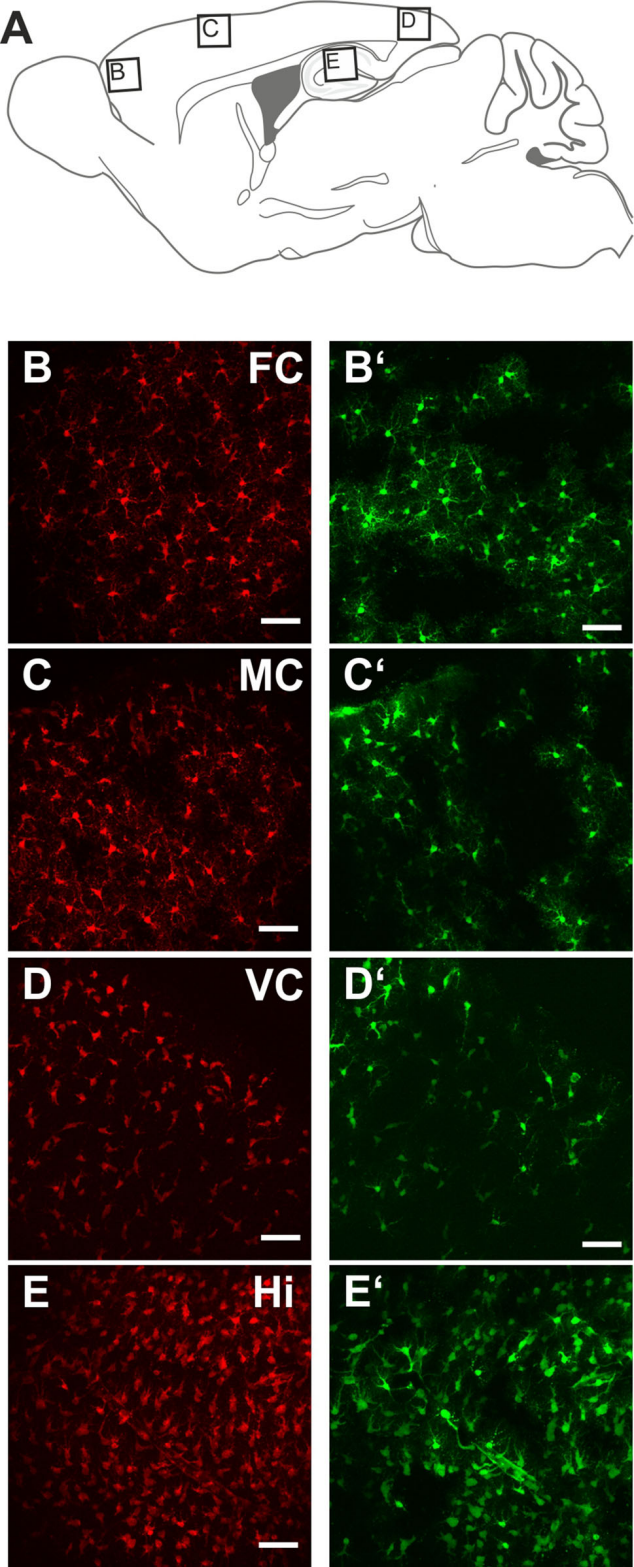
Comparison of SR101 uptake along the rostral–caudal extension of the cortex. **a** Schematic drawing showing rostro-caudal position of images **b**–**e** (*panel* **a** was adapted and modified from the Paxinos Atlas (Paxinos et al. 2001). **b**–**e** SR101-labeling as maximal intensity projections of the 3D two-photon scan (100 μm *z*-stack, 51 sections) from **b** the frontal associative cortex (*FC*), **c** motor cortex (*MC*) and **d** visual cortex (*VC*). Scans were taken from parasagittal slices (P10, 1–2 mm lateral to the midline). Additionally, a maximal intensity projection image from the hippocampus (*Hi*) is depicted in (**e**). **b**′–**e**′ The corresponding EGFP expression is shown next to the SR101 image.* Scale bars* 40 μm

In an initial set of experiments, we compared SR101 staining from different regions of the brainstem, midbrain and hippocampus using [TgN (hGFAP-EGFP)] mice at ages between P8 and P23. The SR101 intensity in all the tested regions of the brainstem was weak, especially when compared with the labeling in the hippocampus (Fig. 1). There was some regional heterogeneity also within the brainstem, with more intense labeling in the lateral superior olive (LSO; Fig. 1k), but altogether the SR101-labeling of EGFP-positive astrocytes was far below the level in hippocampal EGFP-positive cells. Additionally, we tested SR101-labeling along the rostral–caudal extension of the cortex of sagittal slices (Fig. 2). The astroglial SR101-labeling at the three tested cortical areas was indistinguishable from each other. Thus, this test proved that our approach of sorting EGFP-positive astrocytes from hippocampus (Hi) (and cortex; Cx) and brainstem will be valid to detect a putative transporter for SR101.

### Differential expression of *Oatp1c1* in astrocytes from forebrain over brainstem

**Fig. 3:**
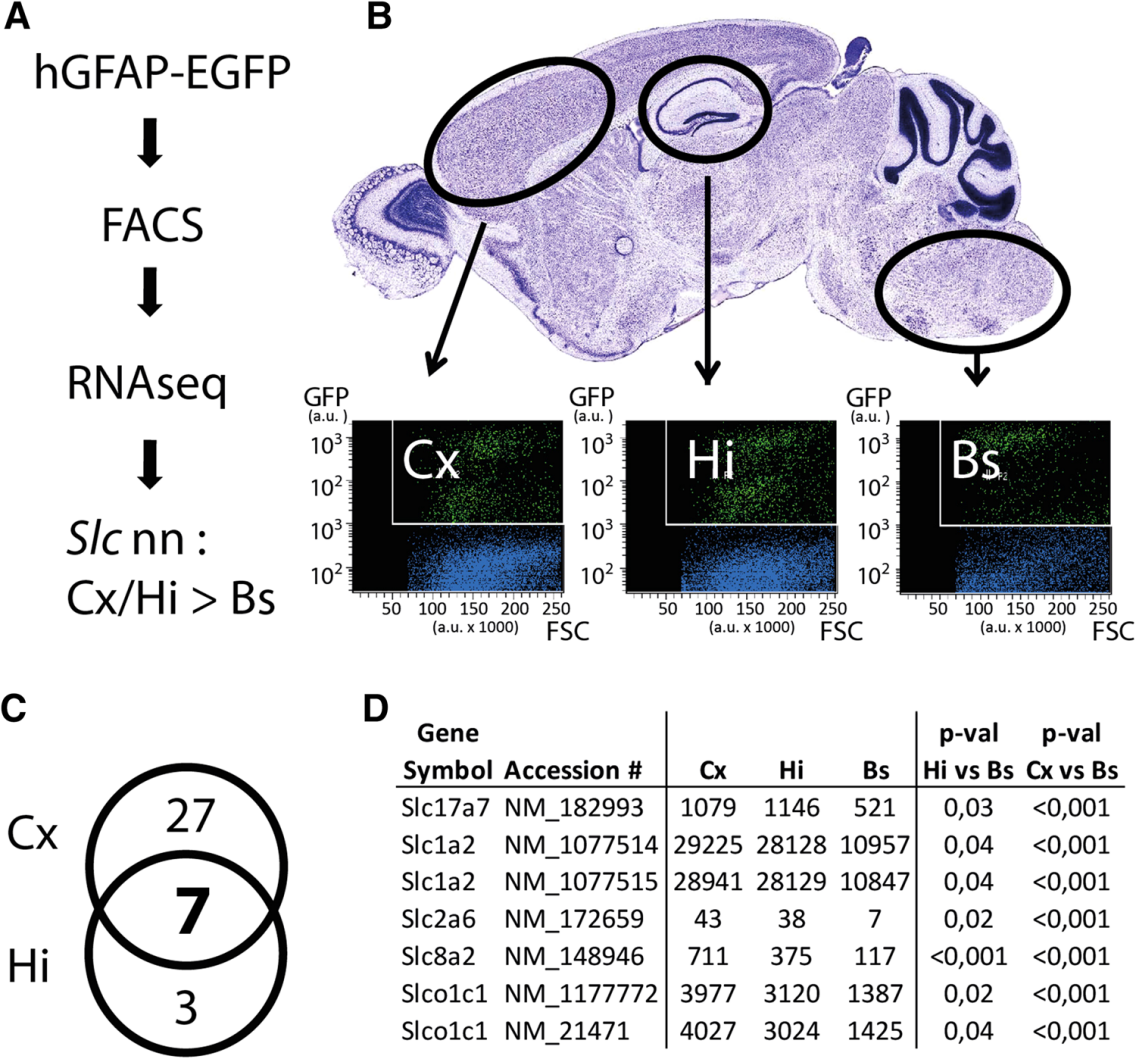
Identification of transporter genes enriched in forebrain astrocytes by transcriptome analysis. **a** Experimental strategy: cortex (*Cx*), hippocampus (*Hi*) and brainstem (*Bs*) tissues from brains of mice expressing EGFP under the control of the glial-specific promoter GFAP (*hGFAP-EGFP*) were triturated and 50 k EGFP-positive cells were purified by FACS, followed by RNA isolation and transcriptome profiling with RNAseq. SR101 candidate uptake transporter genes (of the solute carrier, Slc family) were hypothesized to display a higher expression in Cx and Hi versus Bs astrocytes (*Cx/Hi >Bs*). **b** Scatter plots of FACS analysis with GFP intensities plotted versus forward scatter (*FSC*) given as arbitrary units (*a.u.*). *Blue dots* represent Hoechst 33,342 positive and GFP negative cells, *green dots* represent Hoechst 33,342 and GFP double-positive cells representing different astrocyte populations. **c** Venn diagram of all forebrain enriched transporter genes. 27 (*Cx*) and 3 (*Hi*) refseq annotated mRNAs were detected to be significantly higher expressed in Cx or Hi astrocytes (>2 fold change), and seven mRNAs were significantly elevated both in Cx and Hi. **d** Differentially expressed mRNAs coding for solute carriers (*Slc* and *Slco* gene families). The gene symbols, accession and average read numbers in Cx, Hi and Bs samples are depicted. The seven detected forebrain enriched mRNAs correspond to five genes (*Slc1a2* and *Slco1c1* annotated by two mRNAs each). Detection cutoff was determined with an adjusted *p* value cutoff <0.05. Cx (*n* = 2), Hi (*n* = 3), Bs (*n* = 4) biological replicates

Based on the hypothesis that the putative SR101 transporter belongs to the solute carrier (organic anion transporter) family (Schnell et al. 2012), we focused the analysis of our next-generation sequencing results on these genes (slc or slco gene symbols). Comparing the mRNA levels of astrocytes from hippocampus (Hi), cortex (Cx) and brainstem (BS), we identified 359 transporter mRNAs (mapped refSeq IDs, see methods) coding for 276 proteins that were expressed at least in one of the three different regions (≥10 reads, see supplementary table). However, we detected only seven mRNAs encoding for five transporters of solute carrier families including the glial glutamate transporter 1 (*Glt1/Slc1a2*), the sodium calcium exchanger (*Ncx2/Slc8a2*) and the vesicular glutamate transporter 1 (*Vglut1/Slc17a7*) that were expressed higher in the forebrain (Cx and Hi) vs. brainstem astrocytes (Fig. 3d). There was no organic anion transporter (OAT; *Slc22* family) among these genes, but from the *Slco* family, we found mRNAs for two isoforms of the organic anion transporting polypeptide (*Oatp*)*1c1* (*Slco1c1*) that were differentially expressed between forebrain and brainstem astrocytes (>2 fold increase, adjusted *p* value <0.05). Additionally there were ten genes that showed higher mRNA levels in the brainstem as compared to the Cx and Hi, including the known astroglial transporters for inhibitory neurotransmitter, GAT3 (encoded by *Slc6a11*) and GlyT1 (encoded by *Slc6a9*; supplementary table.)

### Thyroid hormone inhibition of SR101-labeling in hippocampal astrocytes

**Fig. 4:**
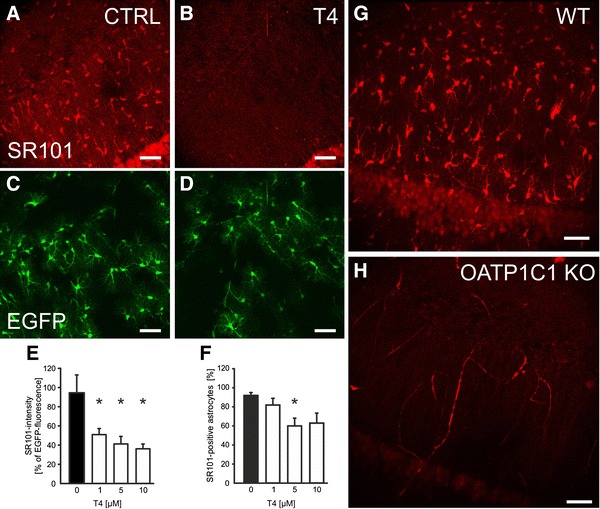
The thyroid hormone transporter OATP1C1 (SLCO1C1) is the astrocytic uptake transporter for SR101. **a**–**d** Maximal intensity projections of SR101-labeling hippocampus slices from hGFAP-EFGP mouse (P22) under control conditions (*CTRL* **a**) and in the presence of 10 μM levothyroxine during the 20 min loading period (*T4* **b**). Corresponding EGFP fluorescence is shown in *green* below the SR101 figure (**c**, **d**).** e**,** f** The basic statistical description of the blockade of SR101 uptake by T4 is shown in (**e**) for the SR101 intensity as relative to the EGFP fluorescence. **f** Comparison of the fraction of EGFP-positive astrocytes with a SR101-fluorescence signal larger than background (SR101-positive astrocytes). Data are given as mean ± SEM (*n* = 4 slices, four animals; one way ANOVA with multiple comparisons versus Control Group; Bonferroni *t* test). Significance is indicated by an *asterisk* for *p* < 0.05.** g**,** h** SR101 uptake is diminished in *Oatp1c1* knockout mice. While astrocytes were labeled by SR101 in Hi slices from WT mice from the same strain (three animals, *n* = 5 slices), no SR101-labeling was observed in hippocampus slices from Slco1c1 KO mice (five animals; *n* = 15 slices). Note that the unspecific labeling in the superficial neurons of the CA1 pyramidal layer (Thompson et al. 2006) is still present after T4 and in the knockout slices

*Oatp1c1/Slco1c1* has been identified to code for a thyroid hormone transporter(OATP14, OATP-F; Pizzagalli et al. 2002) and, indeed, this candidate was the only one with a very similar pharmacological profile (Sugiyama et al. 2003) as described for the putative SR101 uptake system in hippocampal slices (Schnell et al. 2012). To test if the physiological substrate of OATP1C1, l-thyroxine (T4), can compete with the SR101 uptake, we incubated acute hippocampal slices (P21–P22) in SR101 in the presence of different concentrations of T4 (1–10 μM; *n* = 4). The intensity of SR101 fluorescence in EGFP-labeled astrocytes of the stratum radiatum was significantly reduced in a dose-dependent manner (Fig. 4a–e). The relative number of EGFP-positive cells, which showed some detectable, although very low level of SR101 fluorescence (SR101-positive cells), however, did not follow this dose dependency (Fig. 4f). Taken together, the observed reduction in intensity of SR101-labeling supported our hypothesis that OATP1C1 is the transporter responsible for SR101 uptake into astrocytes.

### Lack of SR101-labeling of astrocytes from Oatp1c1 knockout mice

To further substantiate this assumption that OATP1C1 is the astroglial SR101-uptake transporter, we analyzed SR101 uptake in *Oatp1c1* null mutant (*Slco1c1*−*/*−) mice (Mayerl et al. 2012). In hippocampal slices from *Slco1c1*−*/*− mice, the identification of astrocytes by SR101 is impossible (Fig. 4g–h). Since we could not identify any SR101-labeled cell that morphologically resembled an astrocyte, we further concluded that the weak labeling observed in astrocytes of the ventrolateral medulla (Schnell et al. 2012) could in fact result from the low, albeit robustly detected level of expression of *Oatp14/Slco1c1* in the brainstem compared to hippocampus and cortex (Fig. 3d). In this context, it is interesting to note that the unspecific labeling that we sometimes observed in the superficial neurons of the CA1 pyramidal layer (Fig. 4 and supplementary movie) can still be observed after T4 application (Fig. 4b) and in the knockout slices (Fig. 4h).

## Discussion

### SR101 uptake in hippocampal astrocytes is mediated by *OATP1C1*

**Fig. 5:**
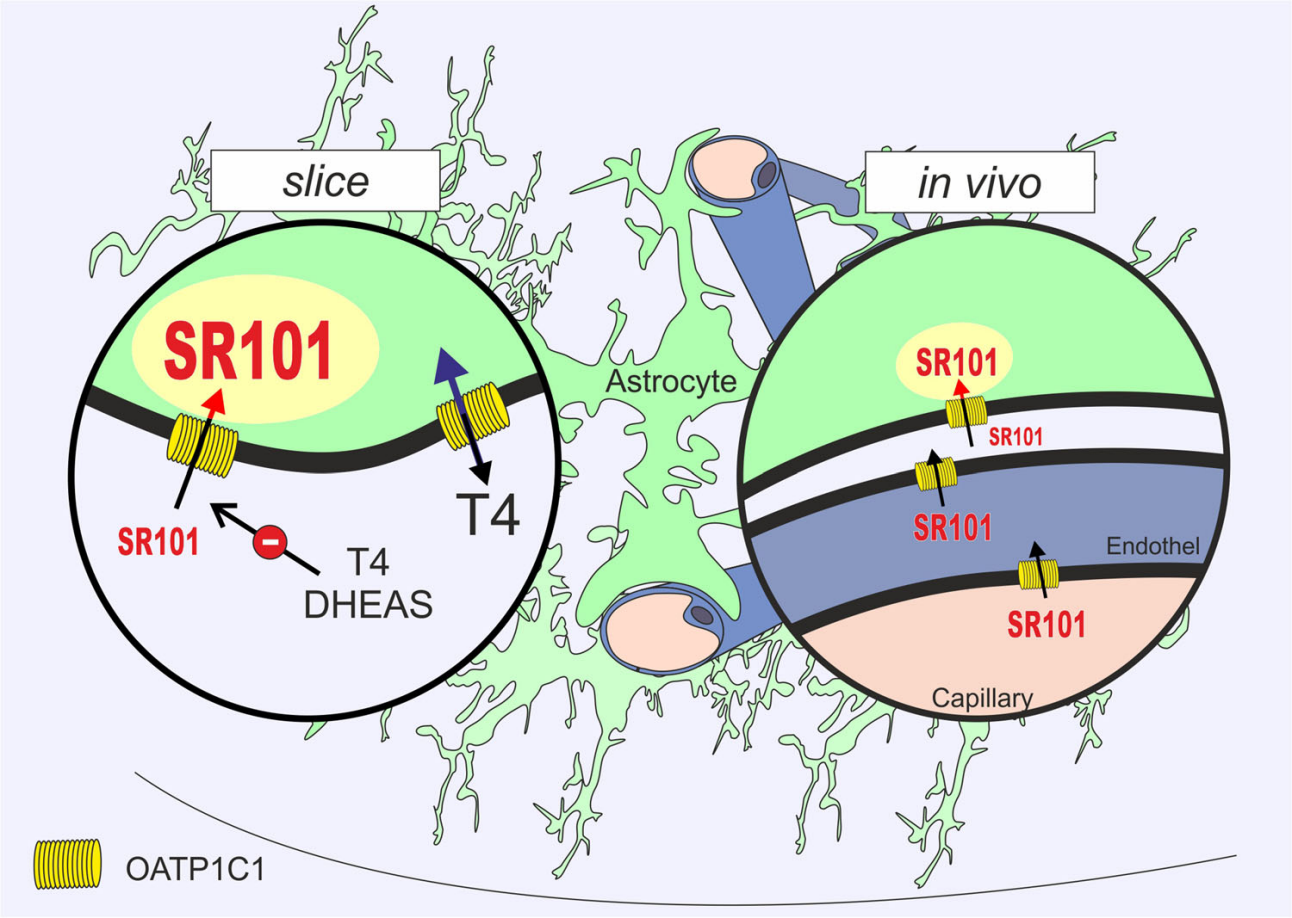
Schematic of the SR101-labeling mechanism. The figure summarizes the mechanism of SR101-labeling in astrocytes. In slices (*left*) SR101 is taken up into astrocytes that express the thyroid hormone transporter OATP1C1 (*Yellow*). Uptake is blocked by levothyroxine (*T4*; see Fig. 4a–f) and neurosteroids e.g., dehydroepiandrosterone sulfate [*DHEAS*; (Schnell et al. 2012)]. Furthermore (*right* in vivo), Oatp1c1 is also expressed on the vascular endothelium (Ridder et al. 2011) allowing uptake of SR101 from the blood stream (Appaix et al. 2012). Since SR101 still labels vessels in the Oatp1c1 knockout mouse (Fig. 4h), there might be a second transporter that transports SR101 into the endothelium. Alternatively, there might be also unspecific binding of SR101 to vasculature, as described, e.g., for Alexa Fluor 633 (Shen et al. 2012)

*Oatp1c1/Slco1c1* cDNA, coding for OATP1C1 that is also known as OATP-F and OATP14, was cloned in 2008 as part of the blood–brain barrier genome program (Chu et al. 2008). This high-affinity transporter for thyroxine (T4) acts sodium-independently in a bidirectional manner (Pizzagalli et al. 2002; Sugiyama et al. 2003; Tohyama et al. 2004). Apart from T4, other OATP1C1 substrates, like probenecid, estrone-3-sulfate (Tohyama et al. 2004) or dehydroepiandrosterone (Sugiyama et al. 2003) reduced SR101 uptake in hippocampal slices (Schnell et al. 2012). Although OATP1C1 expression on the endothelial side of the blood–brain barrier is established, the function of OATP1C1 as a putative astrocytic transporter for T4 uptake is still under debate (Grijota-Martínez et al. 2011; Ridder et al. 2011; Heuer and Visser 2013). Our experiments finally prove the expression of OATP1C1 in subpopulations of astrocytes. Moreover, the overlap of OATP1C1 expression with Aquaporin 4 (Roberts et al. 2008) suggests a preferential targeting of the transporter to astroglial end feet of the blood brain barrier. This polarized expression of OATP1C1 expression on end feet together with the endothelial expression (Fig. 5) explains why SR101 can cross the blood brain barrier and label astrocytes when injected into the vasculature (Appaix et al. 2012). The absence of astroglial SR101-labeling in the Oatp1c1−/− mice suggests that exclusively OATP1C1 mediates astrocytic SR101 uptake in hippocampal astrocytes.

### A general, region-specific heterogeneity of astrocytes

Our next-generation sequencing (NGS) approach not only allows us to identify the uptake transporter for SR01 that explains the region-specific differences of SR101-labeling observed in the cardio-respiratory regions of the brainstem (Schnell et al. 2012), but also sheds some light on a more general heterogeneity of astrocytes. We could detect differences in the expression of neurotransmitter transporters that have already been reported before as for the glycine transporter 1 (*Slc6a9*), GAT3 (*Slc6a11*) or the glial glutamate transporter GLT1 (*Slc1a2*), a finding that underscores the reliability of our method (Supplementary table). Since expression of the transporter apparently correlates with the importance of the different transmitter systems in these brain regions, it is tempting to speculate that differential expression of the OATP1C1 across the forebrain and brainstem is based on a more active involvement of forebrain astrocytes in the control of local thyroid hormone metabolism.

Interestingly, in parallel to the forebrain-enriched expression of the astroglial thyroid hormone transporter Oatp1c1, we also found a higher expression of the type 2 iodothyronine deiodinase (Dio2), the enzyme that converts thyroxine (T4) to 3,3′,5-triiodothyronine (T3), in the cortex and the hippocampus as compared to Bs (Cx 14,564, Hi 8,120, Bs 3,947 norm. reads; adjusted *p* values Cx vs. Bs <0.001 and Hi vs. Bs = 0.0886). Further investigation will be necessary to unveil the relevance of astroglial function and eventually brain development, but the differential expression of OATP1C1 appears to be a rather general difference that is even preserved in humans. Indeed, human *OATP1C1* shows a region-specific expression with preferential detection of mRNA in the forebrain over the hindbrain (Pizzagalli et al. 2002).

### Methodological considerations

As in a preceding paper (Schnell et al. 2012), we observed differences in the significance levels when comparing the reduction of SR101 intensity, which was significant for all T4 concentrations tested, with the apparent numbers of SR101-labeled EGFP-positive astrocytes. Due to the manual detection (see methods), which we found to be superior to the automatic detection, all astrocytes with SR101 intensities that are just above the background are counted and every EGFP-positive astrocyte with an incomplete blockade of the transporter counts as an SR101-positive cell. Therefore, we have to assume that the SR101 uptake by the OATP1C1 is not completely blocked by the applied concentrations of T4. However, in the *Oatp1c1*−/− slice, we were unable to detect individual cell somata in the SR101 channel, suggesting that there was no alternative route of SR101 uptake in hippocampal astrocytes.

Our sequencing experiments were performed on FACS-isolated EGFP-expressing cells from TgN (hGFAP-EGFP) mice. The majority of the EGFP-expressing cells have been proven to be astrocytes in the brain regions from which we took FACS samples (Nolte et al. 2001; Grass et al. 2004). When interpreting the mRNA data, however, one must be aware that the FACS-sorted EGFP-positive cells from the hippocampus might include some neural precursors, e.g., from the subgranular zone of the dentate gyrus, which are also EGFP positive (Hüttmann et al. 2003). Additionally, the EGFP-labeled cells population also includes a subpopulation of NG2-positive cells (oligodendrocyte precursor cells) that also were shown to be EGFP-positive in the hippocampus (Matthias et al. 2003; Jabs et al. 2005) as well as in the brainstem (Grass et al. 2004; Szöke et al. 2006). For our aim, the identification of the SR101 transporter, however, contamination with non-astrocyte cell populations was not critical, since we confirmed these results with pharmacological experiments and by using the *Slco1c1* knockout mouse.

### Unspecific neuronal labeling in the CA1-pyramidal layer

In some of our experiments, we noticed unspecific labeling of pyramidal neurons close to the slice surface (see Fig. 3 and supplementary movie). Such unspecific neuronal labeling has been reported before, although using different labeling conditions, including lower temperature (Nimmerjahn and Helmchen 2012) or reduced extracellular magnesium concentration (Thompson et al. 2008) or hypoxia (Thompson et al. 2006). From the observation that unspecific staining was also found after application of T4 or in the OATP1C-deficient situation (Fig. 4), one might conclude that the unspecific neuronal staining results from another mechanism that is not available or active in the astrocyte. To answer the question whether this involves the opening of pannexin hemichannels, as suggested (Thompson et al. 2006, 2008), further experiments are requires that are beyond the scope of the paper.

### Perspectives

Although our study primarily aimed at identifying the uptake mechanism of SR101 into astrocytes that underlies the regional difference of SR101-labeling, we would like to emphasize that by matching pharmacological properties with cell type-specific expression profiles of transporters, other dyes or compounds could be rationally designed and/or identified to selectively target additional cell types of the brain. Moreover, our strategy can pave the way toward a rational design of cell type-selective dyes that may be used for basic research purposes and also in the context of diagnostic and therapeutic approaches for, e.g., gliomas, which often have special expression profiles for *OATPs* (Bronger et al. 2005).

## Conclusion

We have demonstrated that hippocampal astrocytes take up sulforhodamine 101 via the thyroid hormone transporter OATP1C1. This observation will help to understand the possible side effects of SR101 application and prevent pitfalls when using the drug in novel preparations or under modified condition.

## Electronic supplementary material

Below is the link to the electronic supplementary material.

Supplementary material 1 (XLSX 7923 kb)

Supplementary movie: The movie shows a 3 dimensional view of the CA1 stratum radiatum (same data set as depicted in Fig. 4A-D). The left part shows the control (CTRL) situation. For the right experiment 10 μM L-Thyroxine (T4) was co-applied during the SR101 incubation (20 min). SR101 staining (red channel); EGFP-signal (green channel); Volume 400 × 400 × 100 μm³. Note the unspecific labeling of superficial neurons in the pyramidal layer of both, CTRL and T4 experiments. (MOV 61822 kb)
